# Preparedness of healthcare workers for the Ebola outbreak in Mubende and Kassanda districts, Uganda

**DOI:** 10.4102/jphia.v16i4.1347

**Published:** 2025-08-29

**Authors:** Patricia E. Nabwami, Jackline M. Nyaberi, Norah N. Monyangi, Noelina Nantima, Joshua Kayiwa, Aggrey G. Mokaya

**Affiliations:** 1 Kenya Medical Research Institute (KEMRI) Graduate School, Nairobi, Kenya; 2 Department of Environmental Health and Disease Control, School of Public Health, Jomo Kenyatta University of Agriculture and Technology, Juja, Kenya; 3 Department of Public Health Emergency Operations Centre, Ministry of Health, Kampala, Uganda; 4 Department of Public Health and Sanitation, Ministry of Health, Kisumu, Kenya; 5 Food and Agriculture Organisation of the United Nations (FAO), Representation in Sudan, Port Sudan, Sudan; 6 Africa Health Research Institute, Durban, South Africa

**Keywords:** Ebola Virus Disease, disease outbreaks, viral haemorrhagic fevers, public health preparedness, response, Mubende, Kassanda, Uganda

## Abstract

**Background:**

Effective preparedness is essential to safeguard healthcare workers (HCWs) and strengthen outbreak response. The 2022–2023 Ebola virus disease (EVD) outbreak in Uganda exposed critical gaps in healthcare preparedness, with HCWs accounting for 13.4% cases and 12.7% deaths.

**Aim:**

The study assessed preparedness of HCWs in public health facilities in Mubende and Kassanda districts, Uganda for EVD containment.

**Setting:**

The study was conducted in 16 public health facilities in districts severely affected by the 2022–2023 EVD outbreak.

**Methods:**

A cross-sectional study was conducted in May 2024 and June 2024 among 376 HCWs. Preparedness was assessed based on knowledge, infection prevention and control practices (IPC) practices and attitudes towards EVD containment. Data were collected using self-reported structured questionnaires. Preparedness was determined using median split. Logistic regression analysis was performed in STATA, and 95% confidence intervals (CIs) were calculated to assess statistical significance.

**Results:**

One hundred and fifteen (30.6%) HCWs met preparedness criteria. A total of 295 HCWs (78.5%) could not correctly don personal protective equipment (PPE), while 258 (68.6%) could not correctly doff PPE. The HCWs with degree or higher had higher odds of being prepared (adjusted odds ratio [aOR]: 4.55, 95% CI: 1.26–16.45) compared to those with lower qualifications. Similarly, HCWs with 11–15 years of experience had higher odds of being prepared compared to those with fewer years (aOR: 3.47, 95% CI: 1.12–10.07).

**Conclusion:**

Overall preparedness among HCWs was low. This highlights the need for continuous professional development and routine practical training on PPE use including donning and doffing procedures.

**Contribution:**

Findings provide evidence to guide targeted interventions for improving HCW preparedness for future EVD outbreaks.

## Introduction

Ebola Virus Disease (EVD) is a severe infectious disease with mortality rates ranging from 25% to 90%.^1^ Uganda had so far experienced four outbreaks of the Sudan strain^2^ by the time this study was conducted. There is currently no approved vaccine nor treatment for EVD Sudan strain underscoring the vulnerability of Uganda’s healthcare system to future outbreaks.^3^

Lessons from past outbreaks have spurred the Government of Uganda to invest in epidemic preparedness resulting in significant improvement in the country’s response to Ebola.^4^ The Government of Uganda has put in place stringent measures to improve early detection, reporting and rapid response to EVD. These consist of training of healthcare workers (HCWs), community engagement, infection prevention and control (IPC) measures including safe and dignified burials, equipping of EVD isolation and treatment units.^5^

Despite these investments, the 2022/2023 outbreak exposed critical gaps in Uganda’s healthcare system. The outbreak originated from Mubende district and spread to nine other districts in Uganda with a case fatality rate of 47.0%.^6^ Of great concern were infections among HCWs that accounted for 13.4% of total cases and 12.7% of deaths,^7^ highlighting their heightened vulnerability during outbreaks. Recent studies have indicated that HCWs are 21 to 32 times more likely to acquire infectious diseases compared to individuals in the general population.^8^ Therefore, preparedness of HCWs before an epidemic is of paramount importance to ensure their safety.^9^ Failure to strengthen preparedness of HCWs will not only jeopardise their health and well-being but will also compromise the broader healthcare system to effectively combat the spread of the virus.

The study aimed at evaluating preparedness of HCWs for EVD outbreak in public health facilities in Mubende and Kassanda districts in Uganda in 2024.

## Research methods and design

### Study design and setting

A cross-sectional study was conducted among HCWs in 16 public health facilities in Mubende and Kassanda districts in Uganda in May 2024 and June 2024. These facilities included one hospital, three Health Centre IVs and all Health Centre IIIs across the two districts.

Mubende and Kassanda districts were selected because they were the most severely affected by the 2022/2023 EVD epidemic.^10^

Mubende district has one hospital, Mubende Regional Referral Hospital, located in Mubende Municipality. It is among the 17 regional referral hospitals in Uganda. In addition, the district has 10 Health Centre IIIs and 59 Health Centre IIs distributed across two counties and one town council. The district does not have any Health Centre IV facility.

Kassanda district was carved off from Mubende district. The district has three Health Centre IVs, three Health Centre IIIs and 17 Health Centre IIs.

### Study population

This study involved HCWs in Mubende and Kassanda districts. An HCW was defined as one who is in direct or indirect care of patients. For purposes of this study, HCWs were classified into three groups: (1) clinical staff (doctors, nurses, clinical officers and midwives), (2) allied health workers (nutritionists, laboratory technicians and pharmacists) and support staff (mortuary attendants, cleaners, ambulance drivers, burial teams and village health teams, among others).

### Inclusion criteria

The study included HCWs who worked in these healthcare facilities during the outbreak. This criterion assumed that HCWs included in the study were substantially familiar with the health facility and its operating protocols. It also included HCWs who provided written consent for the study.

### Exclusion criteria

Healthcare workers who were on leave or absent from the health facilities during this outbreak, as well as those who didn’t give a written consent, were excluded from the study.

### Independent variables and dependent variables

Study variables were categorised into independent and dependent variables. The independent variables included individual HCW-level variables. These variables included the respondent’s sex, age, level of education, job designation, years of experience in the medical field, among others. The study’s dependent variables included HCWs’ knowledge of EVD, availability and access to personal protective equipment (PPEs), knowledge on proper use of PPEs, participation in outbreak management training, attitudes towards managing the disease, among others. In this study, preparedness was defined as the ability of an HCW to respond effectively to an EVD outbreak.

### Sample size and sampling techniques

The sample size was estimated using Kish-Leisle formula for cross-sectional studies. Assuming a 95% confidence interval (CI) and a proportion of HCWs with the highest IPC adherence of 0.43,^11^ a sample size of approximately 376 HCWs was calculated, which was adequate to provide a power of 80%.

A stratified sampling design was adopted to select HCWs who participated in the study. The HCWs were stratified according to their job designation, ensuring representation across different roles within the health facilities. To implement this, a list of eligible HCWs within each job designation category was compiled. Each stratum was then randomly sampled to select participants, employing simple random sampling within each stratum.

### Data collection tools

Data from HCWs were collected using a self-reported structured questionnaire developed by the researcher. The questionnaire was developed based on a review of existing literature, including previous EVD studies conducted in Uganda and Ghana^12,13^ as well as guidelines and materials from Centers for Disease Control and Prevention (CDC) website. No pilot study was conducted.

The structured questionnaire captured data on participants’ socio-demographic characteristics, knowledge on EVD, current IPC practices, willingness to attend to EVD patients and participation in outbreak management training programmes, among others.

### Data management

Completed hard copy questionnaires were manually entered into Kobo Toolbox for electronic capture. The data from Kobo Toolbox were then exported to Microsoft® Excel for cleaning and coding. Continuous variables were grouped into defined categories. Categorical variables were coded numerically for analysis. In cases where missing data were identified, we cross-checked with the original hard copies and, where necessary, revisited the field to retrieve missing information.

### Data analysis

Statistical analysis was performed using STATA version 17 statistical software.

An 18-item questionnaire was used to assess the level of preparedness for EVD among HCWs.

Clinical staff and allied health workers responded to all 18 questions. For support staff, one question relating to knowledge on the incubation period of EVD was not applicable, and therefore they answered 17 questions.

For every correct sequence of answer, one was assigned and for every wrong sequence of answers, zero was assigned. For data presentation, results were coded as ‘Yes’ to represent correct responses (1) and ‘No’ to represent incorrect responses (0).

A median split approach was used to categorise HCWs as prepared or unprepared. Those scoring above median were classified as prepared and those below as unprepared. This method has been used in similar studies assessing preparedness levels.^14^

The median split for clinical staff and allied health workers was different from the support staff because the former were expected to be deeply knowledgeable about EVD compared to the latter who had basic knowledge. For the clinical staff and allied health worker, the lowest and highest scores were seven and 18, respectively, with a median of 13. A median split was done at a score of ≤ 13 for unprepared and > 13 for prepared.

For support staff, the lowest and highest scores were 2 and 15, respectively. The median split was done at ≤ 12 for unprepared and > 12 for prepared. Descriptive statistics for categorical variables were reported as frequencies and percentages. For continuous variables, medians and interquartile ranges (IQR) were calculated.

Logistic regression was done to establish the association between independent variables and level of preparedness. Crude odds ratios (cORs) were computed for each independent variable. Variables with *p*-values < 0.2 at bivariable analysis were used in the multivariable analysis. At the multivariable analysis, interaction and confounding were assessed. Adjusted odds ratios (aOR) were reported for each independent variable.

Confidence interval was used to determine statistical significance and ascertain the precision of various estimates.

### Ethical considerations

The proposal and data collection tools were submitted to the research ethics committee of ‘The AIDS Support Organization’ (TASO) in Uganda and approval was granted. Final approval and research licence were issued by Uganda National Council for Science and Technology with a research registration number of SS2828ES. Written permission to visit health facilities was given by District Health Officers. Written informed consent was obtained from study participants and Key informants emphasising voluntary participation. Participants’ identities were not recorded, and all data were anonymised. Only authorised personnel accessed the data, with regulatory bodies reviewing information solely for compliance, without compromising privacy. The study participants were given a moderate compensation of 10 000 Uganda shillings.

## Results

### Social demographic characteristics of healthcare workers

Overall, Mubende district constituted 265 (70.5%) of the participants, and Kassanda districts constituted 111 (29.5%) participants. Clinical staff accounted for 218 (58.0%) of the participants, 157 (41.8) were aged between 30 years and 39 years, 167 (44.4%) held diplomas, 264 (70.2%) were permanently employed and 145 (38.5%) had between 5 years and 10 years of work experience (Table 1).

**TABLE 1:** Social demographic characteristics of health workers.

Characteristics of healthcare workers	Overall		Kassanda		Mubende	
	*n*	%	*n*	%	*n*	%
**District**						
Kassanda	111	29.5	-	-	-	-
Mubende	265	70.5	-	-	-	-
**Sex**						
Female	197	52.4	56	50.5	141	53.2
Male	179	47.6	55	49.5	124	46.8
**Level of health facility**						
Health Centre III	160	42.5	43	38.7	117	44.2
Health Centre IV	68	18.1	68	61.3	0	0.0
Regional Referral Hospital	148	39.4	0	0.0	148	55.8
**Age (years)**						
18–29	78	20.7	22	19.8	56	21.1
30–39	157	41.8	47	42.3	109	41.5
40–49	108	28.7	36	32.4	72	27.2
≥ 50	33	8.8	6	5.4	27	10.2
**Designation**						
Support staff	99	26.3	28	25.2	71	26.8
Clinical staff	218	58.0	61	55.0	157	59.2
Allied health worker	59	15.7	22	19.8	37	14.0
**Level of education**						
Certificate	143	38.0	52	46.8	91	34.3
Degree and above	46	12.3	4	3.7	42	15.9
Diploma	167	44.4	52	46.8	115	43.4
Secondary or less	20	5.3	3	2.7	17	6.4
**Nature of employment**						
Permanent	264	70.2	85	76.6	179	67.5
Temporary	91	24.2	23	20.7	68	25.7
Volunteer	21	5.6	3	2.7	18	6.8
**Years of experience**						
Less than 5 years	124	33.0	28	25.2	96	36.2
5–10 years	145	38.5	49	44.2	96	36.2
11–15 years	77	20.5	29	26.1	48	18.1
Over 15 years	30	8.0	5	4.5	25	9.5

### Ebola preparedness among healthcare workers

Overall, the study revealed that only one-third of HCWs were prepared to effectively respond to a potential EVD outbreak. The study found that 275 (73.1%) of HCWs had participated in training programmes related to EVD. Regarding PPE use, 295 (78.5%) of study participants reported incorrect sequence of donning and 258 (68.6%) reported incorrect sequence of doffing. Additionally, 288 (76.6%) of HCWs indicated unwillingness to attend to EVD-suspected patients, and 240 (63.8%) reported unwillingness to go into isolation if they developed symptoms of EVD (Table 2).

**TABLE 2:** Descriptive summary of Ebola preparedness among healthcare worker.

Characteristics of healthcare workers	Overall		Kassanda		Mubende	
	*n*	%	*n*	%	*n*	%
**Have you heard of Ebola Virus Disease?**						
No	2	0.5	0	0.0	2	0.8
Yes	374	99.5	111	100.0	263	99.2
**What are the symptoms of Ebola Virus Disease?**						
No	12	3.2	5	4.5	7	2.6
Yes	364	96.8	106	95.5	258	97.4
**How is Ebola Virus Disease transmitted?**						
No	23	6.1	1	0.9	22	8.3
Yes	353	93.9	110	99.1	243	91.7
**What is the incubation period of EVD?**						
No	213	56.6	79	71.2	134	50.6
Yes	77	20.5	15	13.5	62	23.4
NA	86	22.9	17	15.3	69	26.0
**What PPEs are used during handling Ebola-suspected patients?**						
No	31	8.2	10	9.0	21	7.9
Yes	345	91.8	101	91.0	244	92.1
**Select the correct sequence of donning PPEs?**						
No	295	78.5	76	68.5	219	82.6
Yes	81	21.5	35	31.5	46	17.4
**Select the correct sequence of doffing PPEs?**						
No	258	68.6	92	82.9	166	62.6
Yes	118	31.4	19	17.1	99	37.4
**Do you have access to the PPEs listed above?**						
No	27	7.2	5	4.5	22	8.3
Yes	349	92.8	106	95.5	243	91.7
**How accessible are the PPEs at your health facility?**						
No	123	32.7	33	29.7	90	34.0
Yes	253	67.3	78	70.3	175	66.0
**Are you aware that a person suspected of having EVD has to be isolated?**						
No	7	1.8	0	0.0	7	2.6
Yes	369	98.1	111	100.0	258	97.4
**Are you willing to attend to an EVD suspected patient?**						
No	288	76.6	93	83.8	195	73.6
Yes	88	23.4	18	16.2	70	26.4
**What is your level of confidence in handling an EVD suspected patient?**						
No	296	78.7	87	78.4	209	78.9
Yes	80	21.3	24	21.6	56	21.1
**Do you know what to do if you have symptoms of Ebola Virus Disease?**						
No	6	1.6	0	0.0	6	2.3
Yes	370	98.4	111	100.0	259	97.7
**Would you agree to be isolated if you have signs and symptoms of Ebola Virus Disease?**						
No	240	63.8	70	63.1	170	64.2
Yes	136	36.2	41	36.9	95	35.8
**Do you know how to report a potential EVD case?**						
No	11	2.9	1	0.9	10	3.8
Yes	365	97.1	110	99.1	255	96.2
**Are you concerned about the risk of contracting EVD in the course of your duties?**						
No	10	2.7	0	0.0	10	3.8
Yes	366	97.3	111	100.0	255	96.2
**To what extent do you think that adherence to IPC measures can minimise the risk?**						
No	5	1.3	0	0.0	5	1.9
Yes	371	98.7	111	100.0	260	98.1
**Have you participated in any training courses focused on EVD in the past 1 year?**						
No	101	26.9	10	9.0	91	34.3
Yes	275	73.1	101	91.0	174	65.7
**Which of the following training have you received regarding Ebola containment?**						
No	102	27.1	11	9.9	91	34.3
Yes	274	72.9	100	90.1	174	65.7
**Preparedness among healthcare workers**						
No	261	69.4	79	71.2	182	68.7
Yes	115	30.6	32	28.8	83	31.3

EVD, Ebola Virus Disease; PPE, personal protective equipment; IPC, infection prevention and control.

### Factors associated with preparedness of healthcare workers

Healthcare workers with a degree and above had 4.55 times higher odds of being prepared for an EVD outbreak (aOR: 4.55, 95% CI: 1.26–16.45) compared to those with lower qualifications. Similarly, those with diploma had 2.87 times higher odds of being prepared (aOR: 2.87, 95% CI: 1.06–7.76) as compared to those with lower qualifications.

In addition, having 11–15 years of work experience was associated with 3.47 times higher odds of preparedness for EVD (aOR: 3.47, 95% CI: 1.12–10.07) (Table 3).

**TABLE 3:** Factors associated with Ebola Virus Disease preparedness among healthcare workers in Mubende and Kassanda districts in Uganda.

Characteristics of healthcare workers	Crude OR	95% CI	*p*	Adjusted OR	95% CI	*p*
**District**						
Kassanda	Ref	-	-	-	-	-
Mubende	1.13	0.69–1.83	0.632	-	-	-
**Level of health facilities**						
Health Centre III	Ref	-	-	-	-	-
Health Centre IV	1.00	0.54–1.87	0.996	-	-	-
Regional Referral Hospital	1.15	0.71–1.87	0.562	-	-	-
**Sex**						
Female	Ref	-	-	Ref	-	-
Male	1.37	0.88–2.13	0.162	1.44	0.90–2.32	0.128
**Age**						
18–29	Ref	-	-	-	-	
30–39	1.72	0.93–3.17	0.082	-	-	-
40–49	1.25	0.64–2.43	0.510	-	-	-
≥ 50	1.16	0.46–2.93	0.747	-	-	-
**Designation**						
Support staff	Ref	-	-	-	-	-
Clinical staff	0.93	0.56–1.55	0.777	-	-	-
Allied health worker	0.78	0.38–1.59	0.492	-	-	-
Certificate	Ref	-	-	Ref	-	-
**Level of education**						
Degree and above	2.53	1.27–5.04	0.008	4.55	1.26–16.45	**0.021**
Diploma	1.28	0.78–2.10	0.320	2.87	1.06–7.76	**0.038**
Secondary or less	0.31	0.07–1.39	0.125	1.03	0.19–5.61	0.977
**Nature of employment**						
Permanent	Ref	-	-	Ref	-	-
Temporary	0.64	0.37–1.10	0.105	0.64	0.33–1.25	0.193
Volunteer	0.63	0.22–1.76	0.374	0.54	0.17–1.74	0.303
**Years of experience**						
Less than 5 years	Ref	-	-	Ref	-	-
5–10 years	1.34	0.78–2.28	0.287	1.65	0.59–4.57	0.338
11–15 years	1.64	0.89–3.04	0.113	3.47	1.12–10.07	**0.022**
Over 15 years	1.23	0.51–2.97	0.641	2.36	0.55–10.04	0.246

Note: Bold values are significant *p* < 0.05.

OR, odds ratio; CI, confidence interval; Ref, reference.

## Discussion

The study evaluated preparedness of HCWs for EVD outbreaks in Mubende and Kassanda districts in Uganda. Overall, the findings revealed low preparedness among HCWs.

Healthcare workers with higher academic qualifications were significantly more prepared for EVD than those with lower qualifications. This could have been attributed to a deeper understanding of the subject matter. These findings align with recent evidence from a study conducted in Uganda, which demonstrated that health workers with higher education levels exhibited stronger knowledge and adherence to IPC protocols.^15^ It is therefore important for health authorities to promote Continuous Professional Development Programme to create opportunities for HCWs to improve their qualifications.

Additionally, HCWs with more years of work experience were significantly more prepared than those with fewer years of work experience. This could have been attributed to their participation in multiple trainings, which likely enhanced their knowledge and practical skills. Similar patterns were observed among nurses during the coronavirus disease 2019 (COVID-19) pandemic, where those with longer work experience demonstrated significantly higher preparedness compared to those with fewer years of experience.^16^ These findings generally emphasise that extensive work experience contributes to better clinical judgement, familiarity with public health emergencies and a greater ability to interpret and apply guidelines. It is therefore imperative to establish mentorship programmes to enable less experienced HCWs to learn from the institutional knowledge and hands-on expertise of their more experienced colleagues.

Notably, a significant proportion of HCWs were unable to correctly don and doff PPE. This could have been attributed to inadequate regular practical training of the HCWs. A study conducted in Nigeria found out that HCWs were unable to correct don and doff after receiving only video-based trainings without hands-on-practice.^17^ Another study conducted in Bangladesh highlighted that many HCWs lacked confidence and procedural accuracy in PPE usage, particularly during doffing, often because of insufficient hands-on training and unclear guidelines.^18^ These deficiencies are concerning, given that improper PPE usage significantly increases the risk of nosocomial infections, especially during high-risk procedures.^19^ It is therefore important that regular simulation exercises and hands-on-trainings be incorporated as a core component of IPC programmes even outside outbreak periods, to strengthen HCWs competence and confidence in proper PPE use.

The study revealed widespread reluctance among HCWs to attend to suspected EVD patients and to accept isolation if symptomatic. This reluctance may have been because of fear of infection and limited confidence in protective measures. A study in Ghana found that HCWs were often hesitant to isolate themselves or treat EVD patients because of fear, stigma and perceived risks to personal and family safety.^13^ All these findings suggest that fear of contagion and lack of trust in safety measures can significantly undermine outbreak preparedness. Strengthening risk communication and providing psychosocial support are essential to increase HCWs’ confidence and willingness to participate actively in outbreak response efforts.

This study also evaluated the role of support staff, including cleaners, ambulance drivers and burial teams among others in EVD preparedness. Although these individuals are not directly involved in clinical care, they perform crucial tasks such as waste disposal management, patient transportation and implementation of safe burial practices, all of which place them at significant risk of exposure. Their critical involvement in outbreak response underscores the importance of including them in preparedness strategies that extend beyond clinical personnel. Integrating support staff into preparedness efforts ensures that they are equipped with the necessary knowledge and PPE to carry out their duties safely. A previous study from Uganda also emphasised the importance of including support staff in IPC training programmes and ensuring their access to appropriate PPEs as a more holistic and effective response to outbreaks.^20^

Study limitation: The study relied on self-reported data from HCWs rather than direct observation of preparedness practices. This could have introduced a response bias with participants overestimating or underestimating their preparedness.

In conclusion, overall preparedness among HCWs in Mubende and Kassanda districts was found to be low, as only 30.6% of participants were classified as prepared. To enhance the capacity of HCWs to effectively prepare for future EVD outbreaks and other emerging threats, targeted interventions are essential. These should include academic advancement opportunities, structured mentorship programmes, regular hands-on simulation exercises and psychosocial support mechanisms.
